# Prevalence of and factors associated with advanced HIV disease among newly diagnosed people living with HIV in Guangdong Province, China

**DOI:** 10.1002/jia2.25642

**Published:** 2020-11-22

**Authors:** Hongbo Jiang, Jun Liu, Zhimin Tan, Xiaobing Fu, Yingqian Xie, Kaihao Lin, Yao Yan, Yan Li, Yi Yang

**Affiliations:** ^1^ Department of Epidemiology and Biostatistics School of Public Health Guangdong Pharmaceutical University Guangzhou China; ^2^ Department of HIV/AIDS Control and Prevention Guangdong Provincial Center for Disease Control and Prevention Guangzhou China

**Keywords:** people living with HIV, advanced HIV disease, risk perception, social support, provider‐initiated testing and counselling, associated factors

## Abstract

**Introduction:**

A high proportion of people living with HIV (PLHIV) present for care with advanced HIV disease (AHD), which is detrimental to “90‐90‐90” targets to end AIDS by 2030. This study aimed to explore the prevalence of and factors related to AHD among newly diagnosed PLHIV in Guangdong Province, China.

**Methods:**

Newly diagnosed PLHIV were recruited from six cities in Guangdong Province from May 2018 to June 2019. AHD was defined as an initial CD4 count <200 cells/µL or an AIDS‐defining event within one month of HIV diagnosis. Data from a questionnaire and the national HIV surveillance system were used to explore the potential factors related AHD.

**Results:**

A total of 400 of 997 newly diagnosed PLHIV were defined as having AHD with a proportion of 40.1%. After adjusting for statistically significant variables in univariate analysis, multivariable logistic regressions showed that individuals aged 30 to 39 years (adjusted odds ratio (aOR) = 1.77, 95% confidence interval (CI): 1.13 to 2.79) and ≥50 years (aOR = 1.98, 95% CI: 1.15 to 3.43) were at a higher risk of AHD than those aged 18 to 29 years. Participants diagnosed by voluntary counselling and testing (VCT) clinics were less likely to have AHD (aOR = 0.67, 95% CI: 0.48 to 0.94) than those diagnosed at medical facilities. Participants who had ever considered HIV testing (aOR = 0.66, 95% CI: 0.45 to 0.98) and who had high social support (aOR = 0.73, 95% CI: 0.55 to 0.97) were at a lower risk of AHD, whereas participants who had HIV‐related symptoms within one year before diagnosis were at a higher risk of AHD (aOR = 2.09, 95% CI: 1.58 to 2.77). The most frequent reason for active HIV testing was “feeling sick” (42.4%, 255/601), and the main reason for never considering HIV testing was “never thinking of getting HIV” (74.0%, 542/732).

**Conclusions:**

Low‐risk perception and a lack of awareness of HIV‐related symptoms resulted in a high proportion of AHD in Guangdong Province, especially among the elderly, those diagnosed at medical facilities and those with low social support. Strengthening AIDS education and training programmes to scale up HIV testing through provider‐initiated testing and counselling in medical facilities and VCT could facilitate early HIV diagnosis.

## 1 INTRODUCTION

Antiretroviral therapy (ART) has made tremendous progress in reducing morbidity and mortality among people living with human immunodeficiency virus (PLHIV). HIV has been treated as a chronic manageable disease rather than an acute fatal one due to increased access to ART [1]. However, the Joint United Nations Programme on HIV/Acquired immunodeficiency syndrome (UNAIDS) estimated that approximately one out of three PLHIV presented for care with advanced HIV disease (AHD) [2], which was defined as an initial CD4 cell count below 200 cells/μL or an AIDS‐defining event regardless of CD4 cell count according to the European consensus definition [3]. The initial CD4 cell count at HIV diagnosis among PLHIV did not increase significantly from 1992 to 2011 in developed countries [4], which suggested that late HIV diagnosis was not ameliorated. A recent systematic review and meta‐analysis summarized that 34.87% of PLHIV presented for care with AHD in 101 studies [5]. AHD could result in late ART initiation and contribute to inferior treatment outcomes and increased HIV transmission risk [6, 7].

To maximize the benefits of treatment and reduce HIV transmission, in 2015, the World Health Organization (WHO) guidelines recommended treatment for all PLHIV regardless of CD4 cell count [8]. Nevertheless, the decline in HIV‐related mortality seems to have plateaued in recent years, which can be explained by a substantial number of individuals presenting for care and initiating ART with AHD [9, 10]. One recent study suggested that median CD4 cell counts at ART initiation among PLHIV in different income groups generally remained below 350 cells/μL in 2015 [11]. Therefore, it is vital to reduce the proportion of individuals with AHD to facilitate 90‐90‐90 targets and end AIDS by 2030 [2].

A large number of studies have investigated the factors associated with AHD to help identify vulnerable populations with AHD [12]. However, most of the existing literature has investigated demographic and structural factors associated with AHD, such as age, marital status, income, and transmission route using data extracted from the national HIV surveillance system [12, 13]. Limited information is known about psychosocial factors and self‐reported reasons for AHD using data beyond that provided in the surveillance system in China. One previous study conducted in Liuzhou city showed that notifying the spouse/sexual partners of PLHIV about their HIV serostatus promptly and starting provider‐initiated HIV testing and counselling (PITC) in medical facilities were beneficial to early HIV diagnosis [14]. Some psychosocial factors, such as social support, social capital and depression were associated with AHD [15, 16, 17]. Late testing due to the lack of risk perception of HIV infection and awareness of HIV also played an important role in presentation with AHD [18].

Therefore, we conducted this cross‐sectional study to recruit newly diagnosed PLHIV in Guangdong Province to comprehensively explore the factors associated with AHD.

## 2 METHODS

### 2.1 Settings and participants

Guangdong Province is one of the most developed provinces of China and is located on the south‐eastern coast of China; it had a population of more than 113 million in 2018. Guangdong Province ranked fifth in terms of the greatest number of PLHIV in China, and it was one of the 12 provinces reporting more than 10000 PLHIV by the end of 2014 [19]. Six cities were selected as study settings according to the geographical location and economic status. Shantou city in eastern Guangdong Province; Yunfu city in northern Guangdong Province; Yangjiang city in western Guangdong Province; and Zhuhai city, Jiangmen city, and Huizhou city in the Pearl River Delta (the most developed area in Guangdong Province) were selected for the recruitment of participants. Newly diagnosed PLHIV from May 2018 to June 2019 who met the inclusion criteria were recruited using consecutive sampling. The inclusion criteria were as follows: (1) 18 years of age or older; (2) currently living in Guangdong Province; (3) newly diagnosed and reported between May 2018 and June 2019; and (4) able to comprehend the study objectives and procedures and to provide written informed consent. With an average prevalence of AHD in PLHIV (*P*) of 33% [2], a precision error (*d*) of 0.1 *P*, and a confidence level of 95%, the required sample size was calculated to be 858, assuming a non‐response rate of 10%. The staff in six Centers for Disease Prevention and Control (CDC) sites were trained and ready to answer any questions about the survey. They made appointments with the newly diagnosed PLHIV to inform them about their confirmed HIV‐positive result. Participants were invited to complete a questionnaire after signing written informed consent.

### 2.2 Measurements

Data from a self‐designed questionnaire and the national HIV surveillance system were collected in this study. The questionnaire was self‐administered and included items assessing active HIV testing, ever having considered HIV testing, history of facility‐based HIV testing within one year before diagnosis, history of HIV self‐testing, HIV‐related symptoms within one year before diagnosis, medical insurance, social capital, social support and depression. Facility‐based HIV testing within one year before diagnosis referred to testing in voluntary counselling and testing (VCT) clinics, before surgery, blood transfusion and donation, invasive examination (colonoscopy, gastroscope, lumbar puncture, etc.), and so on. Sociodemographic information (age, sex, education, marital status, employment, etc.) and other HIV related information (transmission route, sample source, CD4 cell count, etc.) were extracted from the national HIV surveillance system. Sample sources from medical facilities mainly referred to testing before surgery, blood transfusion and donation, invasive examination, premarital and prenatal examination, sexually transmitted disease (STD) clinics, etc. Sample sources from VCT clinics referred to testing in VCT clinics. Data from the questionnaire and the national HIV surveillance system were matched by the unique infectious diseases reporting card identification (ID).

### 2.3 HIV‐related symptoms

Participants were asked to report any HIV‐related symptoms within one year before diagnosis [14] such as unexplained weight loss, recurrent respiratory tract infections, recurrent cough/chest distress, recurrent oral ulcers, unexplained chronic diarrhoea, unexplained lymphadenectasis, herpes zoster rash, angular cheilitis and hairy leucoplakia, abnormal symptoms of the urethra/genitals, and pulmonary tuberculosis (TB). HIV‐related symptoms were paraphrased into colloquial expressions to make them easier to understand.

### 2.4 Social capital

Social capital is defined as features of social organization that improve the efficiency of society by facilitating coordinated actions. Social capital consists of social trust, social participation, social cohesion and collective engagement [16, 20, 21]. The items used for social capital were retrieved from the Southeastern Pennsylvania Household Health Survey (SPHHS) [16]. Social trust was measured by a single‐item question (“If you lost a wallet or purse that contained RMB 1000 yuan and it was found by a neighbour, do you think it would be returned with the money in it or not?”). Collective engagement was measured by a single‐item question (“Have people in your neighbourhood ever worked together to improve the neighbourhood?”). Social participation was measured by a single‐item question (“How many local groups or organizations in your neighbourhood do you currently participate in, such as social, political, religious, school‐related, or athletic organizations?”). A response to this question containing one or more options was classified as “yes” for social participation. Social cohesion was assessed by four items. The following three items used a 4‐point scale ranging from 1 (strongly disagree) to 4 (strongly agree): (1) “I feel that I belong and am a part of my neighbourhood,” (2) “Most people in my neighbourhood can be trusted” and (3) “People in my neighbourhood are willing to help their neighbours with routine activities such as picking up their trash cans.” One item used a 5‐point scale ranging from 1 (never) to 5 (always): “How often are most people in your neighbourhood willing to help their neighbour?” No validated cut‐off value for social cohesion was available; thus referring to the previous studies, we applied the median as a cut‐off value [22, 23]. The median score for social cohesion was 9 (interquartile range, IQR: 8 to 10); scores ≥ 9 indicated high social cohesion.

### 2.5 Social support

Social support was assessed using the Social Support Rating Scale (SSRS) [24]. The items were rated on a 4‐point scale ranging from 1 to 4. A composite score was obtained by summing the responses to the ten items (Cronbach’s α = 0.66). The median score for social support was 32 (IQR: 27 to 37); scores ≥32 indicated high social support.

### 2.6 Depression

The Center for Epidemiologic Studies‐Depression (CES‐D) 20‐item scale [25] was used to measure depression. The items were rated on a 4‐point scale from 0 to 3, yielding total scores of between 0 and 60 (Cronbach’s α = 0.93). Scores ≥18 indicated depression.

### 2.7 Advanced HIV disease

AHD was defined as an initial CD4 cell count below 200 cells/μL or an AIDS‐defining event regardless of CD4 cell count within one month of HIV diagnosis according to the consensus definition proposed by the European Late Presenter Consensus Working Group [3].

### 2.8 Statistical analysis

The mean and standard deviation were used to present the descriptive results for normally distributed variables, and median and IQR were used for non‐normally distributed variables. Univariate and multivariable non‐conditional logistic regression analyses were performed to explore the factors associated with AHD among the newly diagnosed PLHIV. The statistically significant variables in the univariate analyses were included in the multivariable logistic regression models to compute the adjusted odds ratios (aORs) with 95% confidence intervals (CIs). The difference in the proportion of selected items between groups was compared using χ^2^ tests. All hypothesis tests were 2‐tailed with *α* = 0.05. Data analyses were performed using SAS version 9.4. (SAS Institute Inc., Cary, NC, USA).

### 2.9 Ethical considerations

This study was approved by the Ethics Committee of Guangdong Pharmaceutical University. No incentive was offered to the participants. They were asked to sign a written informed consent document. Participants under formal education signed a written informed consent document after the staff at the local CDC explained the objectives and procedures of the study to them.

## 3 RESULTS

**Figure 1:**
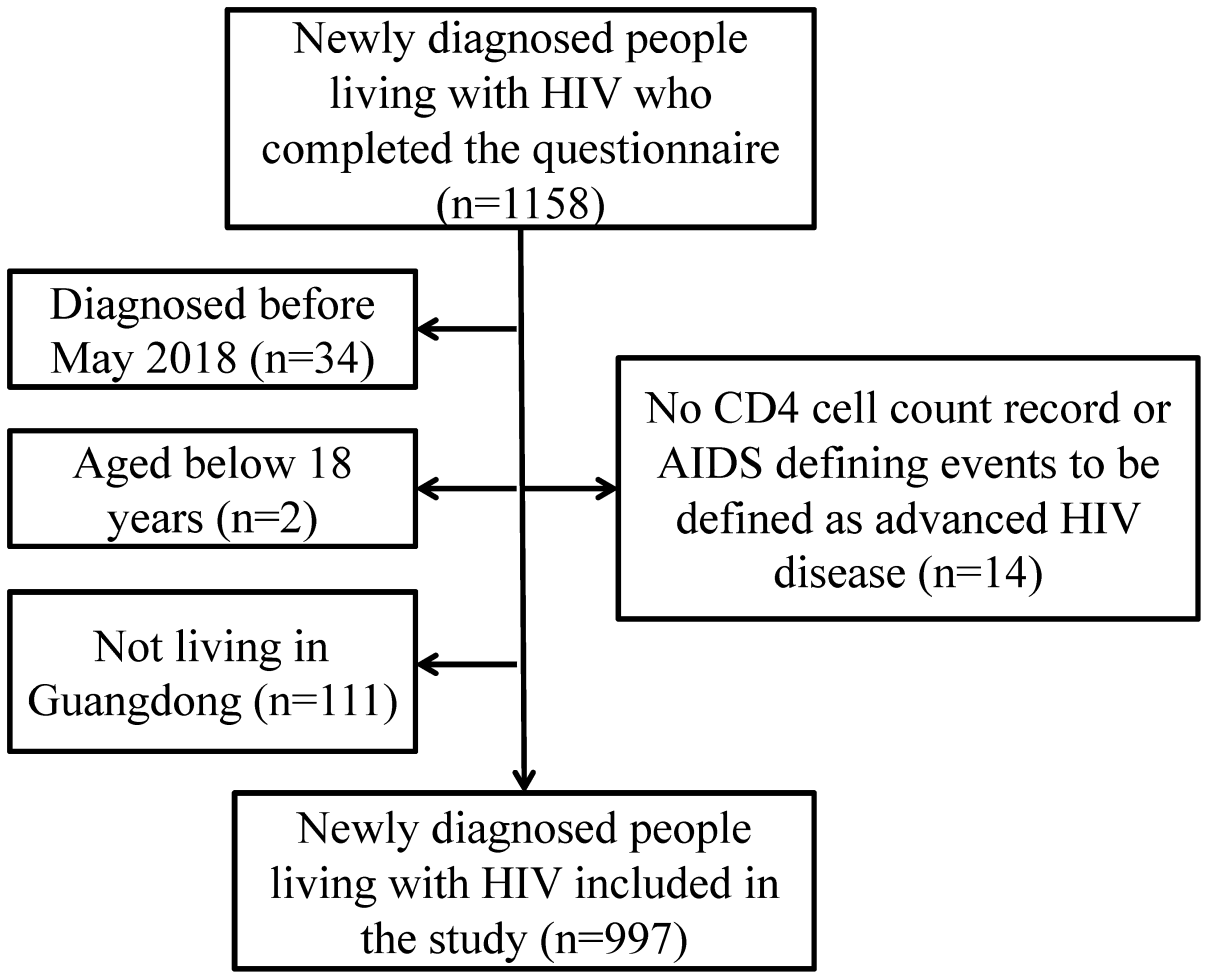
Flow of recruitment and enrollment of newly diagnosed people living with HIV in Guangdong Province, China

A total of 1158 newly diagnosed PLHIV completed the questionnaire, of whom 997 met the inclusion criteria and were included in this study after they matched the cases reported to the national HIV surveillance system (Figure 1). Four hundred cases were defined as AHD with a proportion of 40.1% (400/997). The median age at diagnosis was 44.3 (IQR: 30.7 to 56.0) years, ranging from 18 to 92 years. The majority of the participants were males (80.1%), those with a high school education or below (86.1%), and those with medical insurance (91.5%). Approximately seven in 10 participants experienced transmission by heterosexual contact (67.3%) and were reported from medical facilities (68.5%). The proportion of individuals with active HIV testing, those who considered undergoing HIV testing, those with a history of facility‐based HIV testing within one year before diagnosis and those with a history of HIV self‐testing were 60.3%, 26.6%, 12.4% and 13.6% respectively. Regarding social capital, the proportions of social trust, social participation, collective engagement and high social cohesion were 39.4%, 8.2%, 17.8% and 66.0% respectively. The proportions of depression and high social support were 37.1% and 54.7% respectively. More than 40% of the participants reported any HIV‐related symptoms within one year before diagnosis (Table 1).

**Table 1:** Univariate and multivariable logistic regression analyses on the associated factors with advanced HIV disease among 997 newly diagnosed people living with HIV in Guangdong Province, China

Variable	N (%)	AHD (%)	Crude OR (95% CI)	*p*	Adjusted OR (95% CI)^a^	*p*
Sex						
Male	799 (80.1)	315 (39.4)	1			
Female	198 (19.9)	85 (42.9)	1.16 (0.84 to 1.58)	0.368		
Age at diagnosis (years)						
18 to 29	226 (22. 7)	55 (24.3)	1		1	
30 to 49	412 (41.3)	172 (41.7)	2.23 (1.55 to 3.20)	<0.001	1.77 (1.13 to 2.79)	0.013
≥50	359 (36.0)	173 (48.2)	2.89 (2.00 to 4.18)	<0.001	1.98 (1.15 to 3.43)	0.014
Education						
Primary school or below	286 (28.7)	131 (45.8)	1		1	
Middle school	401 (40.2)	165 (41.1)	0.83 (0.61 to 1.12)	0.225	1.22 (0.86 to 1.74)	0.271
High school or equivalent	171 (17.2)	62 (36.3)	0.67 (0.46 to 0.99)	0.046	1.28 (0.80 to 2.06)	0.307
College or above	139 (13.9)	42 (30.2)	0.51 (0.33 to 0.79)	0.002	1.17 (0.68 to 2.03)	0.566
Marital status						
Unmarried	337 (33.8)	107 (31.8)	1		1	
Married	431 (43.2)	185 (42.9)	1.62 (1.20 to 2.18)	0.002	1.07 (0.71 to 1.63)	0.745
Divorced/widowed	229 (23.0)	108 (47.2)	1.92 (1.36 to 2.71)	<0.001	1.15 (0.73 to 1.80)	0.554
Employment						
No	411 (41.2)	170 (41.4)	1			
Yes	586 (58.8)	230 (39.2)	0.92 (0.71 to 1.18)	0.503		
Medical insurance						
No	85 (8.5)	31 (36.5)	1			
Yes	912 (91.5)	369 (40.5)	1.18 (0.75 to 1.88)	0.473		
Monthly income (yuan)						
0	226 (22.7)	97 (42.9)	1			
1 to 2999	324 (32.5)	142 (43.8)	1.04 (0.74 to 1.46)	0.833		
≥3000	447 (44.8)	161 (36.0)	0.75 (0.54 to 1.04)	0.082		
Transmission route						
Heterosexual contact	671 (67.3)	300 (44.7)	1		1	
MSM	276 (27.7)	80 (29.0)	0.51 (0.37 to 0.68)	<0.001	0.82 (0.54 to 1.23)	0.328
Other	50 (5.0)	20 (40.0)	0.82 (0.46 to 1.48)	0.518	0.75 (0.40 to 1.40)	0.371
Sample source						
Medical facilities^b^	683 (68.5)	308 (45.1)	1		1	
VCT clinics^c^	255 (25.6)	78 (30.6)	0.54 (0.40 to 0.73)	<0.001	0.67 (0.48 to 0.94)	0.019
Other	59 (5.9)	14 (23.7)	0.38 (0.20 to 0.70)	0.002	0.45 (0.24 to 0.87)	0.018
HIV‐related symptoms^d^						
No	427 (42.8)	132 (30.9)	1		1	
Yes	570 (57.2)	268 (47.0)	1.98 (1.52 to 2.58)	<0.001	2.09 (1.58 to 2.77)	<0.001
Active HIV testing						
No	396 (39.7)	183 (46.2)	1		1	
Yes	601 (60.3)	217 (36.1)	0.66 (0.51 to 0.85)	0.002	1.05 (0.77 to 1.43)	0.75
Ever considering HIV testing						
No	732 (73.4)	327 (44.7)	1		1	
Yes	265 (26.6)	73 (27.5)	0.47 (0.35 to 0.64)	<0.001	0.66 (0.45 to 0.98)	0.038
History of facility‐based HIV testing^d^						
No	873 (87.6)	363 (41.6)	1		1	
Yes	124 (12.4)	37 (29.8)	0.60 (0.40 to 0.90)	0.013	0.78 (0.50 to 1.22)	0.277
History of HIV self‐testing						
No	861 (86.4)	369 (42.9)	1		1	
Yes	136 (13.6)	31 (22.8)	0.39 (0.26 to 0.60)	<0.001	0.75 (0.45 to 1.25)	0.271
Social trust						
No	604 (60.6)	260 (43.0)	1		1	
Yes	393 (39.4)	140 (35.6)	0.73 (0.56 to 0.95)	0.02	0.80 (0.60 to 1.07)	0.129
Social participation						
No	915 (91. 8)	371 (40.5)	1			
Yes	82 (8.2)	29 (35.4)	0.80 (0.50 to 1.29)	0.36		
Collective engagement						
No	820 (82.2)	339 (41.3)	1			
Yes	177 (17.8)	61 (34.5)	0.75 (0.53 to 1.05)	0.091		
Social cohesion						
Low	339 (34.0)	149 (44.0)	1			
High	658 (66.0)	251 (38.1)	0.79 (0.60 to 1.03)	0.077		
Social support						
Low	452 (45.3)	198 (43.8)	1		1	
High	545 (54.7)	202 (37.1)	0.76 (0.59 to 0.97)	0.031	0.73 (0.55 to 0.97)	0.029
Depression						
No	627 (62.9)	237 (37.8)	1			
Yes	370 (37.1)	163 (44.1)	1.30 (1.00 to 1.68)	0.052		

AHD, advanced HIV disease; OR (95%CI), odds ratio (95% confidence interval).

^a^ Adjusted for age at diagnosis, education, marital status, transmission route, sample source, HIV‐related symptoms, active HIV testing, ever considering HIV testing, history of facility‐based HIV testing and self‐testing, social trust and social support

^b^ medical facilities: testing before surgery, blood transfusion and donation, invasive examination, premarital and prenatal examination, sexually transmitted disease clinics, etc

^c^ VCT clinics: testing in voluntary counselling and testing clinics

^d^ within one year before diagnosis.

Univariate logistic regression results showed that age at diagnosis, education, marital status, transmission route, sample source, active HIV testing, having ever considered HIV testing, history of facility‐based HIV testing within one year before diagnosis, history of HIV self‐testing, HIV‐related symptoms, social trust and social support were associated with AHD (*p* < 0.05) (Table 1). After adjusting for statistically significant variables in univariate analyses, multivariable logistic regression showed that older individuals were at a higher risk of AHD than those who were younger (30 to 49 vs. 18 to 29: aOR = 1.77, 95% CI: 1.13 to 2.79; ≥50 vs. 18 to 29: aOR = 1.98, 95% CI: 1.15 to 3.43). Sample sources from VCT clinics were less likely to have AHD than those from medical facilities (aOR = 0.67, 95% CI: 0.48 to 0.94). Individuals who had ever considered HIV testing were less likely to have AHD (aOR = 0.66, 95% CI: 0.45 to 0.98), whereas individuals who reported HIV‐related symptoms within one year before diagnosis were more likely to have AHD (aOR = 2.09, 95% CI: 1.58 to 2.77). Individuals with high social support were less likely to have AHD than those with low social support (aOR = 0.73, 95% CI: 0.55 to 0.97) (Table 1).

**Figure 2:**
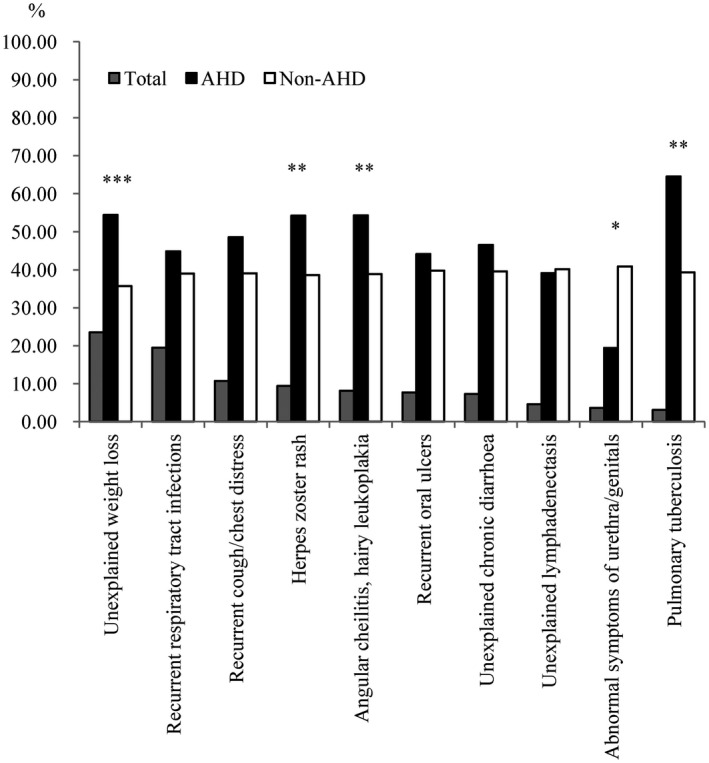
The proportion of HIV‐related symptoms within one year before diagnosis among 997 newly diagnosed people living with HIV in Guangdong Province, China. *P < 0.05, **P < 0.01, ***P < 0.001

**Figure 3:**
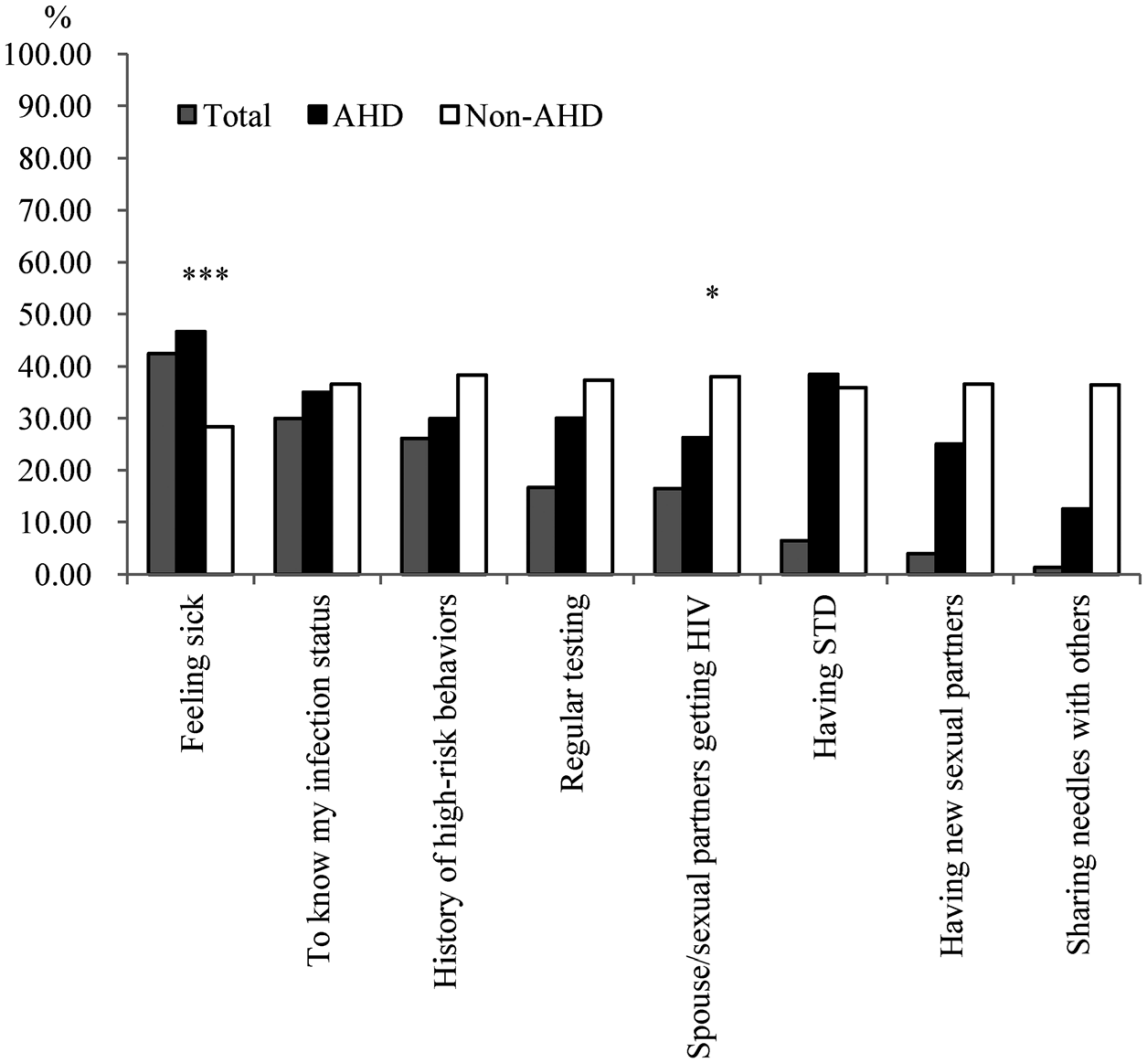
The proportion of reasons for active HIV testing among 601 participants with active HIV testing. *P < 0.05, ***P < 0.001

**Figure 4:**
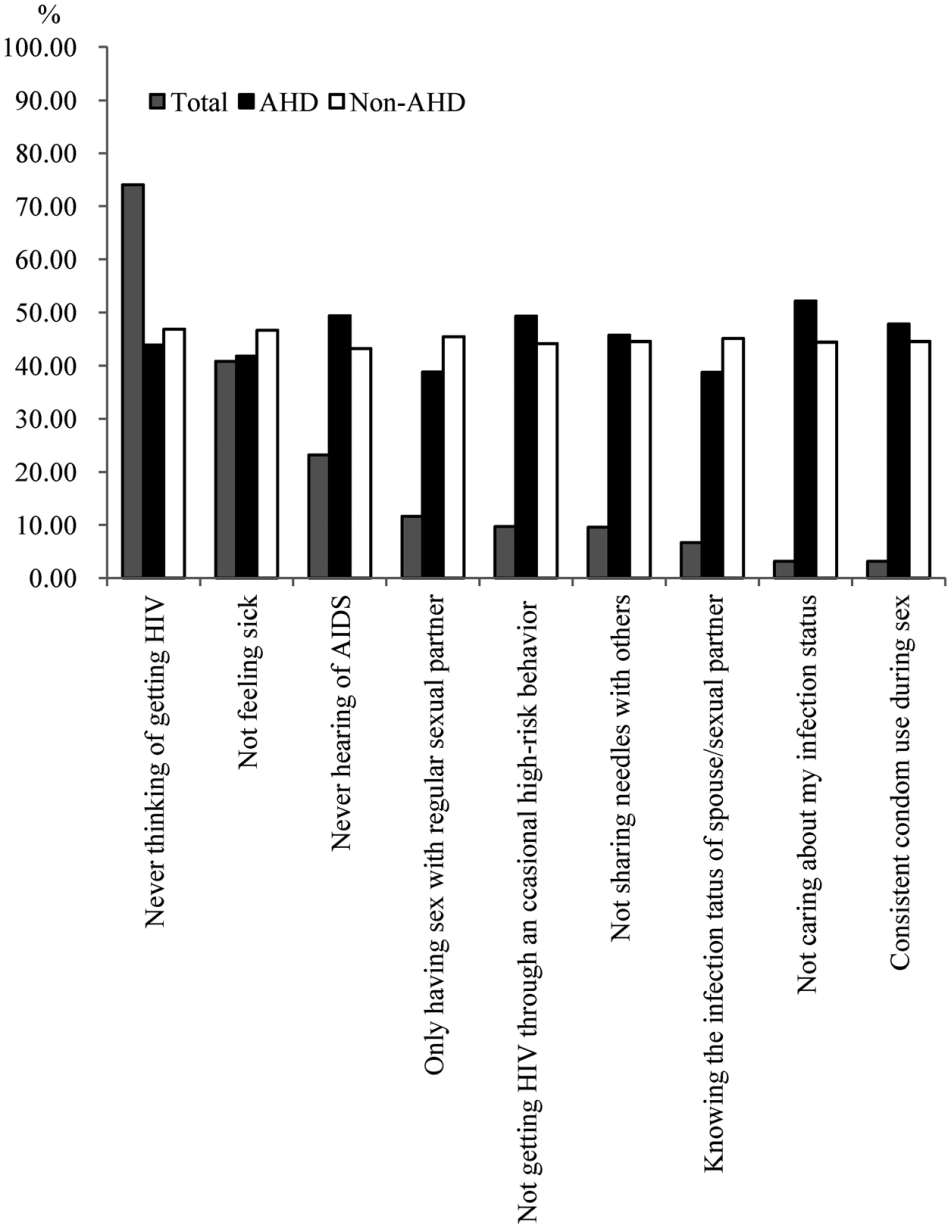
The proportion of reasons for never considering HIV testing among 732 participants who never considered HIV testing.

The most frequent HIV‐related symptom within one year before diagnosis was unexplained weight loss (23.6%), followed by recurrent respiratory tract infections (19.5%). Individuals who presented with AHD reported a higher proportion of unexplained weight loss, herpes zoster rash, angular cheilitis and hairy leucoplakia, and pulmonary tuberculosis, whereas they reported a lower proportion of abnormal symptoms of the urethra/genitals (*p* < 0.05) than those who presented without AHD (Figure 2 and Table S1). Among individuals who reported active HIV testing (n = 601), the most frequent reason was feeling sick (42.2%), followed by desire to know one’s own infection status (30.0%) and a history of high‐risk behaviours (26.1%). With respect to the reasons for active HIV testing, “feeling sick” accounted for a higher proportion among individuals who presented with AHD, whereas “spouse/sexual partners getting HIV” accounted for a lower proportion among individuals who presented with AHD (*p* < 0.05) than among those who presented without AHD (Figure 3 and Table S2). Among individuals who had never considered HIV testing (n = 732), the most frequent reason was “never thinking of getting HIV” (74.0%), followed by “not feeling sick” (40.8%) and “never hearing of AIDS” (23.2%) (Figure 4 and Table S3). Individuals who were reported from VCT clinics had a higher proportion of active HIV testing, having ever considered HIV testing, and a history of HIV self‐testing than those from medical facilities (*p* < 0.001) (Table 2).

**Table 2:** HIV testing and HIV‐related symptoms among participants whose sample sources were medical facilities and voluntary counselling and testing clinics

Variable	N (%)	Sample sources (N = 938)		χ^2^	*P*
		Medical facilities^a^ (%)	VCT clinics^b^ (%)		
HIV‐related symptoms^c^					
No	384 (40.9)	270 (39.5)	114 (44.7)	2.056	0.152
Yes	554 (59.1)	413 (60.5)	141 (55.3)		
Active HIV testing					
No	381 (40.6)	332 (48.6)	49 (19.2)	66.509	<0.001
Yes	557 (59.4)	351 (51.4)	206 (80.8)		
Ever considering HIV testing					
No	685 (73.0)	536 (78.5)	149 (58.4)	38.88	<0.001
Yes	253 (27.0)	147 (21.5)	106 (41.6)		
History of facility‐based HIV testing^c^					
No	816 (87.0)	595 (87.1)	221 (86.7)	0.033	0.856
Yes	122 (13.0)	88 (12.9)	34 (13.3)		
History of HIV self‐testing					
No	806 (85.9)	615 (90.0)	191 (74.9)	35.206	<0.001
Yes	132 (14.1)	68 (10.0)	64 (25.1)		

^a^ Medical facilities: testing before surgery, blood transfusion and donation, invasive examination, premarital and prenatal examination, sexually transmitted disease clinics, etc

^b^ VCT clinics: testing in voluntary counselling and testing clinics

^c^ within one year before diagnosis.

## 4 DISCUSSION

This was one of the few studies conducted in China to comprehensively investigate the factors associated with AHD beyond the data provided in the national HIV surveillance system [14]. The prevalence of AHD in Guangdong Province was high. Older age and HIV‐related symptoms were associated with a higher risk of AHD, while having ever considered HIV testing, sample sources from VCT clinics and high social support were associated with a lower risk of AHD. The most frequent reason for active testing was “feeling sick,” and the main reason for never considering HIV testing was “never thinking of getting HIV.” Our results suggested that strategies should be tailored to target individuals at a high risk of AHD to facilitate early HIV diagnosis.

The prevalence of AHD among newly diagnosed PLHIV in Guangdong Province was relatively higher than that estimated by UNAIDS (one‐third) [2] and lower than that in Guangxi Zhuang Autonomous Region (45.1%) [26]. Although ART should be initiated among everyone living with HIV at any CD4 cell count [8], individuals presenting with AHD may have a lower efficacy of ART and increase the risk of HIV transmission due to a lack of awareness of serostatus [6, 7], which is detrimental to 90‐90‐90 targets and ending AIDS by 2030. Therefore, more efforts should be made to reduce the prevalence of AHD.

Individuals who had ever considered HIV testing may have perceived that they were at high risk of HIV infection. The most frequent reason for never considering HIV testing (“never thinking of getting HIV”) implied the lack of HIV risk perception contributing to AHD. A previous study conducted in Switzerland [18] showed similar results that the main reasons for late HIV testing were that individuals ‘‘did not feel at risk’’ (72%) and ‘‘did not feel ill’’ (65%). Another previous study conducted in 2009 also showed that low initiative for HIV testing and low‐risk perception of HIV infection were the main reasons for late diagnosis in Liuzhou city [14]. Policies in response to the AIDS epidemic have been implemented for years in China including "four free and one care" to expand access to HIV testing and clinical care services, routine HIV testing in sentinel surveillance, and promoting condom use and adequate HIV‐related knowledge in a "five‐year action plan" among the key populations [27, 28]. Low‐risk perception of HIV infection could be associated with low adequate HIV‐related knowledge or insufficient provision of necessary HIV‐related knowledge, and lack of access to HIV testing services [29]. Efforts thus remain to be made to increase the provision of necessary HIV‐related knowledge, scale‐up HIV testing and improve the risk perception of HIV infection.

HIV‐related symptoms reported within one year before diagnosis was associated with a higher risk of AHD [30, 31]. Individuals could be more aware of symptoms related to the genitals, leading them to undergo HIV testing, and participants who were aware of these symptoms accounted for a lower proportion of those with AHD; however, they may not be aware of symptoms less specific to STDs (e.g. weight loss) and thus paid less attention to these symptoms, which may explain the higher proportion of these symptoms among those with AHD. As a result, individuals with low awareness of HIV‐related symptoms and risk perception of HIV infection presented with AHD after the onset of clinical symptoms [32]. The most frequent reason ("feeling sick") for active HIV testing also suggested the low‐risk perception of HIV infection. Individuals with a spouse/sexual partner who were HIV positive could have a high‐risk perception of HIV infection, care more about their health and seek HIV testing quickly when they found out that their partners were HIV positive. Late presentation was associated with unknown serostatus of partners among heterosexuals, and was less likely among MSM who reported a known HIV‐infected partner as an infection source [33, 34]. Partner notification was feasible and has the potential to reduce the risk of HIV infection among the high‐risk population in China [35, 36]. Contact tracing can feasibly expand HIV testing uptake and identify new HIV infections among sexual partners of HIV‐positive individuals [37, 38]. However, stigma/discrimination, fear of negative consequences, and lack of contact information remained barriers to partner notification [35]. Therefore, more efforts to encourage sexual partners of PLHIV to undergo HIV testing may facilitate early HIV diagnosis.

Social support was associated with a low risk of AHD, which may be attributable to good health‐seeking behaviour when people were supported by others [15]. The emotional, informational and appraisal support provided by family members were identified as especially critical for emotional stability, coping and linkage to care during the initial stages of diagnosis and ART [39]. A significantly positive relationship between higher social trust and lower late HIV diagnosis at a state‐level analysis was observed in a previous study [20]. High social capital was associated with increased supportive social norms, higher information exchange and reduced HIV/AIDS stigma, which could result in timely HIV testing and engagement in HIV care [20]. However, little is known about the relationship between social capital and AHD at the individual level. The non‐significant association in the multivariable analysis may be explained by the possible ecological fallacy or the population and culture difference [16, 20, 21]. Thus, items for social capital were suggested to be sinicized and validated before implementation in China. More studies need to be conducted to investigate the relationship between social capital and AHD at the individual level.

Lower risk perception of HIV infection, limited sexual health information targeting the elderly population, less access to health care and missed opportunities in health care settings may result in AHD among older persons [13, 26]. Moreover, the number and proportion of PLHIV among older adults have increased in recent years in China [40]. Individuals initiating ART at an older age showed a poorer immunological response and a higher risk of AIDS‐related death [41], highlighting the urgent need to promote early diagnosis and treatment. Several strategies could be tailored for the elderly individuals to prevent HIV infection and late HIV diagnosis. First, AIDS education campaigns should be continued and strengthened to reach the elderly individuals tailored for their needs to improve the awareness of AIDS and the risk perception of HIV infection. Second, the provision of free condoms and interventions for condom use could facilitate reducing the high‐risk behaviours since the majority of the cases among elderly individuals were mainly sexually transmitted. Third, social support and family support should be provided to meet their physiological and psychological needs. Fourth, HIV testing could be combined to the basic public health services in the community to expand HIV testing coverage among the elderly individuals [42].

Sample sources from VCT clinics were less likely to have AHD than those from medical facilities [13, 14]. VCT clinics could help acquire HIV‐related knowledge and awareness, reduce high‐risk behaviours, and facilitate early referral to care and support [43]. However, participants whose sample sources from VCT clinics accounted for a relatively low proportion, which could be attributable to the fear of stigma and discrimination [44]. This result highlighted the necessity to strengthen the publicity and education about VCT clinics to further improve the use of VCT clinics. PITC in medical facilities aimed to decrease barriers to HIV testing and to increase HIV testing rates and thereby facilitate earlier access to HIV treatment and prevention [45]. However, the most commonly reported barrier to HIV testing by physicians at STD clinics was a low perceived prevalence of disease [46]. The facilitators for acceptance of PITC among MSM in Shenyang were a high awareness of their own risk of HIV infection, having attended a VCT clinic several times and considering PITC beneficial for family and friends [47]. Participants whose sample sources from medical facilities had lower proportions of those with active HIV testing, those who had ever considered HIV testing and those with a history of HIV self‐testing in our study, and these individuals may receive a late diagnosis. Therefore, more efforts including the provision of PITC training for providers and increasing awareness and initiative of HIV testing for participants should be made to improve the acceptance and uptake of PITC and HIV testing coverage.

Our study had several limitations. First, the cross‐sectional design precludes causal inference. Second, although Guangdong is one of the most developed provinces in China, the unbalanced economic status could lead to different characteristics of the AIDS epidemic and proportions of AHD in parts of the province. The non‐probability sampling method was applied because there was no sampling frame for this hard‐to‐reach population, which might contribute to selection bias. Nevertheless, the six cites were selected according to the geographical location and economic status, which may reduce the impact of the selection bias. Although the proportion of AHD could be overestimated due to the downward trend in AHD in Guangdong Province from 51.9% in 2010 to 33.7% in 2016 [48], our results could provide references for settings similar to Guangdong Province with a large number of floating populations at higher risk of HIV infection and the main sexual transmission route of HIV infection [49]. Third, PITC could be one of the key facilitators of early diagnosis, and the awareness and willingness of clinicians to initiatively provide HIV testing also played an important role in strengthening PITC [14]. Therefore, more studies are needed to investigate the facilitators and barriers to PITC to help PLHIV enter care before they present with AHD.

## 5 CONCLUSIONS

The prevalence of AHD in Guangdong was high, especially among individuals who were older, had never considered HIV testing, reported HIV‐related symptoms, had low social support and whose sample sources were from medical facilities. Low‐risk perception of HIV infection and a lack of awareness of HIV‐related symptoms contributed to the high proportion of AHD. The provision of HIV testing and social support for high‐risk populations, especially for the elderly individuals is vital, and strengthening AIDS education and training programmes to scale‐up HIV testing through PITC and VCT should be tailored to facilitate early HIV diagnosis, to ultimately help achieve the 90‐90‐90 targets and end AIDS by 2030.

## COMPETING INTEREST

The authors declare that they have no competing interests.

## AUTHORS’ CONTRIBUTIONS

HJ, YYang and YL designed the research study. JL, ZT, XF, YX, KL, and YYan contributed to the acquisition of data. ZT and HJ analysed and interpreted the data. HJ drafted the manuscript. YYang and YL revised the manuscript critically for important intellectual content. All the authors reviewed and approved the manuscript.

## Supporting information


**Table S1.** HIV‐related symptoms within one year before diagnosis among 997 newly diagnosed people living with HIV in Guangdong Province, China
**Table S2.** Reasons for active HIV testing among 601 participants with active HIV testing in Guangdong Province, China
**Table S3.** Reasons for never considering HIV testing among 732 participants who never considered HIV testing in Guangdong Province, China
