# Supplementary material for: Prevalence of and factors associated with advanced HIV disease among newly diagnosed people living with HIV in Guangdong Province, China
Source: J Int AIDS Soc. 2020 Nov 22;23(11):e25642. doi: 10.1002/jia2.25642 (PMC7680922; doi:10.1002/jia2.25642)
Supplement: Supplementary file 1 — Table S1. HIV‐related symptoms within one year before diagnosis among 997 newly diagnosed people living with HIV in Guangdong Province, China Table S2. Reasons for active HIV testing among 601 participants with active HIV testing in Guangdong Province, China Table S3. Reasons for never considering HIV testing among 732 participants who never considered HIV testing in Guangdong Province, China [file JIA2-23-e25642-s001.docx]

Supplementary Table 1. HIV-related symptoms within one year before diagnosis among 997 newly diagnosed people living with HIV in Guangdong Province, China

| HIV-related symptoms^†^ | N(%) | AHD ^‡^ (%) | Crude *OR*(95%*CI*) ^§^ | *P* |
| --- | --- | --- | --- | --- |
| **Unexplained weight loss with progressive decline >10% in 3 months** | | | |  |
| No | 762 (76.4) | 272 (35.7) | 1.00 |  |
| Yes | 235 (23.6) | 128 (54.5) | 2.16 (1.60 to 2.90) | **<0.001** |
| **Recurrent respiratory tract infections ≥ twice within 6 months** | | | | |
| No | 803 (80.5) | 313 (39.0) | 1.00 |  |
| Yes | 194 (19.5) | 87 (44.8) | 1.27 (0.93 to 1.75) | 0.135 |
| **Recurrent cough/chest distress >1 month** | | |  |  |
| No | 890 (89.3) | 348 (39.1) | 1.00 |  |
| Yes | 107 (10.7) | 52 (48.6) | 1.47 (0.99 to 2.20) | 0.059 |
| **Herpes zoster rash** |  |  |  |  |
| No | 903 (90.6) | 349 (38.6) | 1.00 |  |
| Yes | 94 (9.4) | 51 (54.3) | 1.88 (1.23 to 2.89) | **0.004** |
| **Angular cheilitis, hairy leukoplakia** | |  |  |  |
| No | 916 (91.9) | 356 (38.9) | 1.00 |  |
| Yes | 81 (8.1) | 44 (54.3) | 1.87 (1.19 to 2.95) | **0.007** |
| **Recurrent oral ulcers ≥ twice within 6 months** | | |  |  |
| No | 920 (92.3) | 366 (39.8) | 1.00 |  |
| Yes | 77 (7.7) | 34 (44.2) | 1.20 (0.75 to 1.91) | 0.453 |
| **Unexplained chronic diarrhoea >1 month** | | |  |  |
| No | 924 (92.3) | 366 (39.6) | 1.00 |  |
| Yes | 73 (7.3) | 34 (46.6) | 1.33 (0.82 to 2.15) | 0.244 |
| **Unexplained lymphadenectasis >1 month** | | |  |  |
| No | 951 (95.4) | 382 (40.2) | 1.00 |  |
| Yes | 46 (4.6) | 18 (39.1) | 0.96 (0.52 to 1.76) | 0.889 |
| **Abnormal symptoms of urethra/genitals** | | |  |  |
| No | 961 (96.4) | 393 (40.9) | 1.00 |  |
| Yes | 36 (3.6) | 7 (19.4) | 0.35 (0.15 to 0.80) | **0.014** |
| **Pulmonary tuberculosis** |  |  |  |  |
| No | 966 (96.9) | 380 (39.3) | 1.00 |  |
| Yes | 31 (3.1) | 20 (64.5) | 2.80 (1.33 to 5.92) | **0.007** |

^†^ Self-reported by participants

^‡^ AHD: advanced HIV disease

^§^ OR (95%CI): odds ratio (95% confidence interval)

Supplementary Table 2. Reasons for active HIV testing among 601 participants with active HIV testing in Guangdong Province, China

| Reason | N(%) | AHD ^†^ (%) | Crude *OR*(95%*CI*) ^‡^ | *P* |
| --- | --- | --- | --- | --- |
| **Feeling sick** |  |  |  |  |
| No | 346 (57.6) | 98 (28.3) | 1.00 |  |
| Yes | 255 (42.4) | 119 (46.7) | 2.21 (1.58 to 3.11) | **<0.001** |
| **To know my infection status** | |  |  |  |
| No | 421 (70.0) | 154 (36.6) | 1.00 |  |
| Yes | 180 (30.0) | 63(35.0) | 0.93 (0.65 to 1.35) | 0.712 |
| **History of high-risk behaviors** | |  |  |  |
| No | 444 (73.9) | 170 (38.3) | 1.00 |  |
| Yes | 157 (26.1) | 47 (29.9) | 0.69 (0.47 to 1.02) | 0.062 |
| **Regular testing** |  |  |  |  |
| No | 501 (83.4) | 187 (37.33) | 1.00 |  |
| Yes | 100 (16.6) | 30 (30.00) | 0.72 (0.45 to 1.15) | 0.165 |
| **Spouse/sexual partners getting HIV** | | |  |  |
| No | 502(83.5) | 191 (38.0) | 1.00 |  |
| Yes | 99 (16.5) | 26 (26.3) | 0.58 (0.36 to 0.94) | **0.027** |
| **Having STD** |  |  |  |  |
| No | 562 (93.5) | 202 (35.9) | 1.00 |  |
| Yes | 39 (6.5) | 15 (38.5) | 1.11 (0.57 to 2.17) | 0.752 |
| **Having new sexual partners** | |  |  |  |
| No | 577 (96.0) | 211 (36.6) | 1.00 |  |
| Yes | 24 (4.0) | 6 (25.0) | 0.58 (0.23 to 1.48) | 0.253 |
| **Sharing needles with others** | |  |  |  |
| No | 593(98.7) | 216 (36.4) | 1.00 |  |
| Yes | 8(1.3) | 1 (12.5) | 0.25 (0.03 to 2.04) | 0.195 |

^†^ AHD: advanced HIV disease

^‡^ OR (95%CI): odds ratio (95% confidence interval)Supplementary Table 3. Reasons for never considering HIV testing among 732 participants who never considered HIV testing in Guangdong Province, China

| Reason | N(%) | AHD ^†^ (%) | Crude *OR*(95%*CI*) ^‡^ | *P* |  |
| --- | --- | --- | --- | --- | --- |
| **Never thinking of getting HIV** | |  |  |  |  |
| No | 190 (26.0) | 89 (46.8) | 1.00 |  |  |
| Yes | 542 (74.0) | 238 (43.9) | 0.89 (0.64 to 1.24) | 0.485 |  |
| **Not feeling sick** | | |  |  |  |
| No | 433 (59.2) | 202 (46.7) | 1.00 |  |  |
| Yes | 299 (40.8) | 125 (41.8) | 0.82 (0.61 to 1.11) | 0.195 |  |
| **Never hearing of AIDS** | |  |  |  |  |
| No | 562 (76. 8) | 243 (43.2) | 1.00 |  |  |
| Yes | 170 (23.2) | 84 (49.4) | 1.28 (0.91 to 1.81) | 0.157 |  |
| **Only having sex with regular sexual partner** | | |  |  |  |
| No | 647 (88.4) | 294 (45.4) | 1.00 |  |  |
| Yes | 85 (11.6) | 33 (38.8) | 0.76 (0.48 to 1.21) | 0.250 |  |
| **Not getting HIV through occasional high-risk behavior** | | | |  |  |
| No | 661 (90.3) | 292 (44.2) | 1.00 |  |  |
| Yes | 71 (9.7) | 35 (49.3) | 1.23 (0.75 to 2.01) | 0.410 |  |
| **Not sharing needles with others** | |  |  |  |  |
| No | 662 (90.4) | 295 (44.6) | 1.00 |  |  |
| Yes | 70 (9.6) | 32 (45.7) | 1.05 (0.64 to 1.72) | 0.853 |  |
| **Knowing the infection status of spouse/sexual partner** | | | |  |  |
| No | 683 (93.3) | 308 (45.1) | 1.00 |  |  |
| Yes | 49 (6.7) | 19 (38. 8) | 0.77 (0.43 to 1.40) | 0.391 |  |
| **Not caring about my infection status** | | |  |  |  |
| No | 709 (96.9) | 315 (44.4) | 1.00 |  |  |
| Yes | 23 (3.1) | 12 (52.2) | 1.37 (0.59 to 3.13) | 0.464 |  |
| **Consistent condom use during sex** | | |  |  |  |
| No | 709 (96.9) | 316 (44.6) | 1.00 |  |  |
| Yes | 23 (3.1) | 11 (47.8) | 1.14 (0.50 to 2.62) | 0.756 |  |

^†^ AHD: advanced HIV disease

^‡^ OR (95%CI): odds ratio (95% confidence interval)
